# Morgagnian cataract resulting from a naturally occurring nonsense mutation elucidates a role of CPAMD8 in mammalian lens development

**DOI:** 10.1371/journal.pone.0180665

**Published:** 2017-07-06

**Authors:** Anne K. Hollmann, Insa Dammann, Wiebke M. Wemheuer, Wilhelm E. Wemheuer, Almuth Chilla, Andrea Tipold, Walter J. Schulz-Schaeffer, Julia Beck, Ekkehard Schütz, Bertram Brenig

**Affiliations:** 1 University of Goettingen, Institute of Veterinary Medicine, Goettingen, Germany; 2 University Medical Center Goettingen, Department of Neuropathology, Prion and Dementia Research Unit, Goettingen, Germany; 3 University of the Saarland, Institute of Neuropathology, Homburg, Germany; 4 University of Veterinary Medicine Hannover, Foundation, Department of Small Animal Medicine and Surgery, Hannover, Germany; 5 Chronix Biomedical, Goettingen, Germany; Rush University Medical Center, UNITED STATES

## Abstract

To investigate the genetic basis of hereditary lens opacities we analyzed 31 cases of bilateral congenital cataract in Red Holstein Friesian cattle. A genome-wide association study revealed a significant association on bovine chromosome 7 at positions 6,166,179 and 12,429,691. Whole genome re-sequencing of one case and four relatives showed a nonsense mutation (g.5995966C>T) in the PZP-like, alpha-2-macroglobulin domain containing 8 (*CPAMD8*) gene leading to a premature stop codon (CPAMD8 p.Gln74*) associated with cataract development in cattle. With immunohistochemistry we confirmed a physiological expression of CPAMD8 in the ciliary body epithelium of the eye in unaffected cattle, while the protein was not detectable in the ciliary body of cattle with cataracts. RNA expression of *CPAMD8* was detected in healthy adult, fetal and cataractous lenses.

## Introduction

Cataracts are opacities of the lens present from birth (congenital) or acquired during life and are the main cause of blindness in humans worldwide [1]. The genetic background of the disease is well studied in humans. Until today more than 290 genes and 19 non-gene loci have been associated with cataract development in humans and mice [2]. Besides hereditary cataracts, opacities also occur as part of multisystemic disorders or due to the impact of environmental factors [3]. In cattle knowledge about the etiology of cataract development (due to genetic or environmental factors) is still relatively scarce, even though an incidence of 26% was reported in some herds [4]. Cataracts have already been observed in several cattle breeds, such as Holstein Friesian [5–8], Jersey [9, 10], Hereford [11], Aberdeen Angus [11], Shorthorn [11] and Ayrshire [4]. So far, only one mutation with recessive inheritance has been identified leading to juvenile-onset bilateral incomplete immature nuclear cataract in Romagnola cattle [12].

Many of the identified mutations leading to cataract development in humans and mice affect genes encoding lens crystallins and have been discussed in detail elsewhere [13]. Mutations in other proteins like membrane, cytoskeleton and gap junction proteins, beaded filaments, growth and transcriptional factors are also known to result in cataract development [3, 14]. Recently, C3 and PZP-like, alpha-2-macroglobulin domain-containing 8 gene (*CPAMD8*), a so-far unknown candidate gene for cataract development, was associated with anterior segment dysgenesis (ASD). ASD manifests itself in a spectrum of developmental abnormalities affecting the anterior segment of the eye, including cataract [15]. CPAMD8 belongs to the complement component 3 (C3)/alpha2-macroglobulin (A2M) family [16], whose members are involved in the innate immune system [17, 18]. Except for the association with ASD, knowledge about the biological function of *CPAMD8* is comparatively scanty. The gene was discussed as being associated with Ashkenazi Jewish Crohn’s disease [19] and multiple sclerosis [20] in humans. Regarding eye development, *CPAMD8* expression was observed to be upregulated in the regeneration-incompetent irides of axolotls [21].

Since 2009, congenital cataracts have been increasingly reported in German Red Holstein Friesian (HF) cattle. A total of 31 cases were observed, and they have provided the opportunity to elucidate the molecular cause of the disorder. As a result we have detected a nonsense mutation in *CPAMD8* (g.5995966C>T) leading to a premature stop codon in exon 1 of the gene associated with congenital cataract development in HF cattle.

## Results

### Clinical findings

All four examined cases showed bilateral complete mature cataracts at time of birth. No other obvious ophthalmological anomalies were observed at this timepoint.

**Fig 1:**
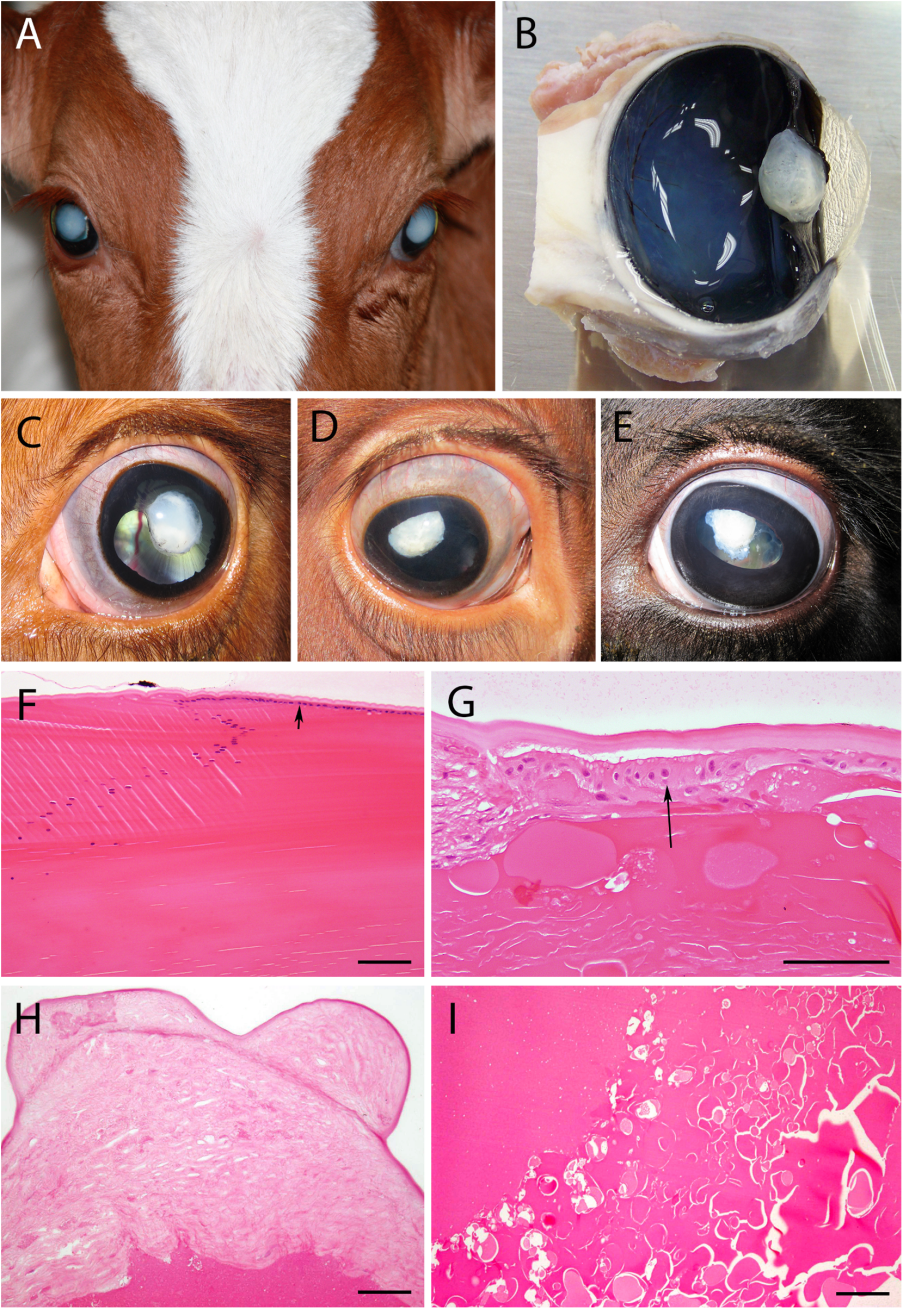
Congenital cataract in Red Holstein Friesian cattle. (A) Bilateral mature cataract formation, (B) sagittal cut through the eyeball (formalin fixed) with cataractous lens (animal #224) clearly showing the irregular lens surface. (C) to (E) are pictures of different stages of the degenerative lenses in situ taken during the clinical examinations: (C) left eye of animal #227 at 13 months, mature cataract, (D) right eye of animal #489 at 31.5 months, mature to hypermature cataract, (E) left eye of animal #908 at 30 months, mature to hypermature cataract. Iris abnormalities as observed by Cheong et al. (2016) in human ASD patients showing cataracts were not detected in bovine cataract cases. (F) to (I) show the histopathological changes in hematoxylin/eosin stained sections (bars = 100μm): (F) lens equatorial line in a bovine healthy adult lens where the anterior epithelium (arrow) under the capsule is clearly visible (control animal). During cataract formation the capsule thickens, the cuboidal epithelium (G, arrow, animal #224, left eye) disintegrates and over time vanishes completely (H, animal #908, left eye). The loss of lens fibrils and presence of Morgagnian globules indicate the presence of a hypermature cataract (G, I, animal #224). Fig 1A was digitally improved and cropped using GIMP 2.8.18.

Eyes of the affected female cattle were examined in detail with the appropriate ophthalmologic equipment at approximately either 13 or 30 months of age (S1 Table). All cases showed a mature to hypermature cataract at time of examination (Fig 1A–1E). Besides the opacities, mydriasis and microphakia (mostly accompanied by an irregular shape of the lens), multiple eye defects like posterior synechia, glaucoma, exophthalmos, uveitis and retinitis (in one case) were additionally observed in some eyes (for details see S1 Table). These anomalies developed during life of cataract cases and were not congenital. The ongoing dissolution of the lens and subsequent inflammatory processes affecting the eye are suspected to be the reason for these clinical findings. In all but one eye the ocular fundus appeared to be free of pathological findings. Anomalies as iris hypoplasia, corectopia and ectropion uveae as observed by Cheong et al. (2016) were not detected. Neurological examination did not show any deficits other than the ones related to the bilateral eye problem.

### Pathology and histopathology

Since the complete eyeballs were fixed in formalin before they were cut for histology, the visual inspection of the inner eye was performed on fixed tissue. Lenses were usually of irregular shape, about two-thirds to half the size of an unaffected lens, and opaque white (Fig 1A–1E). Microphakia was more pronounced in two of the older animals (#908 (Fig 1E) and #489 (Fig 1D), 30 and 31.5 months of age). Histologically, the regular lens would consist of a cuboidal epithelium in its anterior parts (Fig 1F arrow) that turns at the equatorial line, loses its nuclei and cytoplasmatic organelles and degrades to lens fibers, building the bulk of the lens. The lens epithelium usually has a thick basement membrane, forming a capsule (Fig 1F). The cataractous lenses showed a loss of lens epithelium and thickening of the capsule (Fig 1G and 1H), but no clear distinction between basement membrane and connective tissue. The bulk of the lens showed Morgagnian globules (Fig 1G and 1I), liquefactions and mineralizations (not shown) confirming the presence of a hypermature cataract. No fibrillary structures could be observed.

### Autosomal recessive inheritance of congenital cataract formation

**Fig 2:**
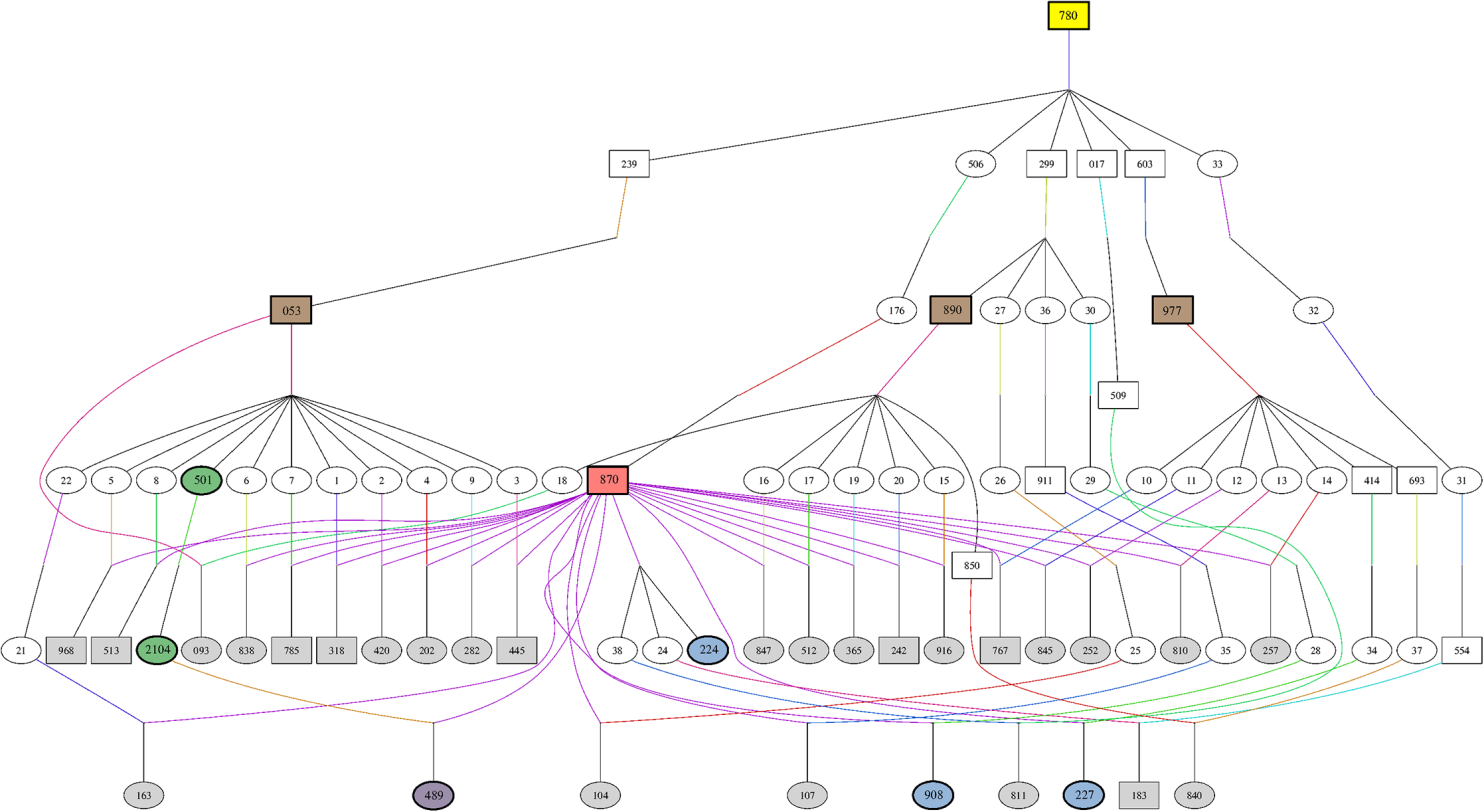
Origin and transmission of cataract in Red Holstein Friesian cattle. The pedigree depicts the ancestry of the affected individuals (grey, purple and blue symbols). Two cases are not displayed in the family tree. Neither maternal nor paternal lines were clearly documented. Animal #183 was also genotyped T/T for the variant g.5995966C>T in *CPAMD8*. The origin of the cataract-causing mutation was traced back to founder sire #780 (yellow symbol). The dam´s sires in 20 of 31 cases were sires #053, #890, #977, marked with brown symbols. The red symbol shows sire #870, father of 26 of 31 cases. This animal (#870) was also used for whole genome re-sequencing, as the green (mother and maternal grandmother of one case) and purple (cattle with congenital cataract) marked cattle. The blue and purple symbols mark the four dissected cases. Family tree was created using Pedigraph [22].

Analysis of the pedigree data revealed that 26 of 31 cases were paternal half-siblings of sire #870 (Fig 2). In 20 of 31 cases, the dams’ fathers were either sires #053, #890 or #977. The four sires #870, #053, #890 and #977 shared one common ancestor (#780) two to three generations before. Sire #780, born in 1982, appeared in 28 of 31 cases in the dam and sire line of the pedigrees. Pedigrees of the remaining three animals were not clearly documented. Parental testing was not performed. However, an autosomal recessive inheritance of the cataract phenotype can be inferred from pedigree analyses, although a relation to the predicted founder (#780) on the dam´s side could not be verified in three cases. Sire #780 was genotyped as a carrier and is most likely the founder of the defect.

### Association and region of extended homozygosity on bovine chromosome 7 (BTA7)

**Fig 3:**
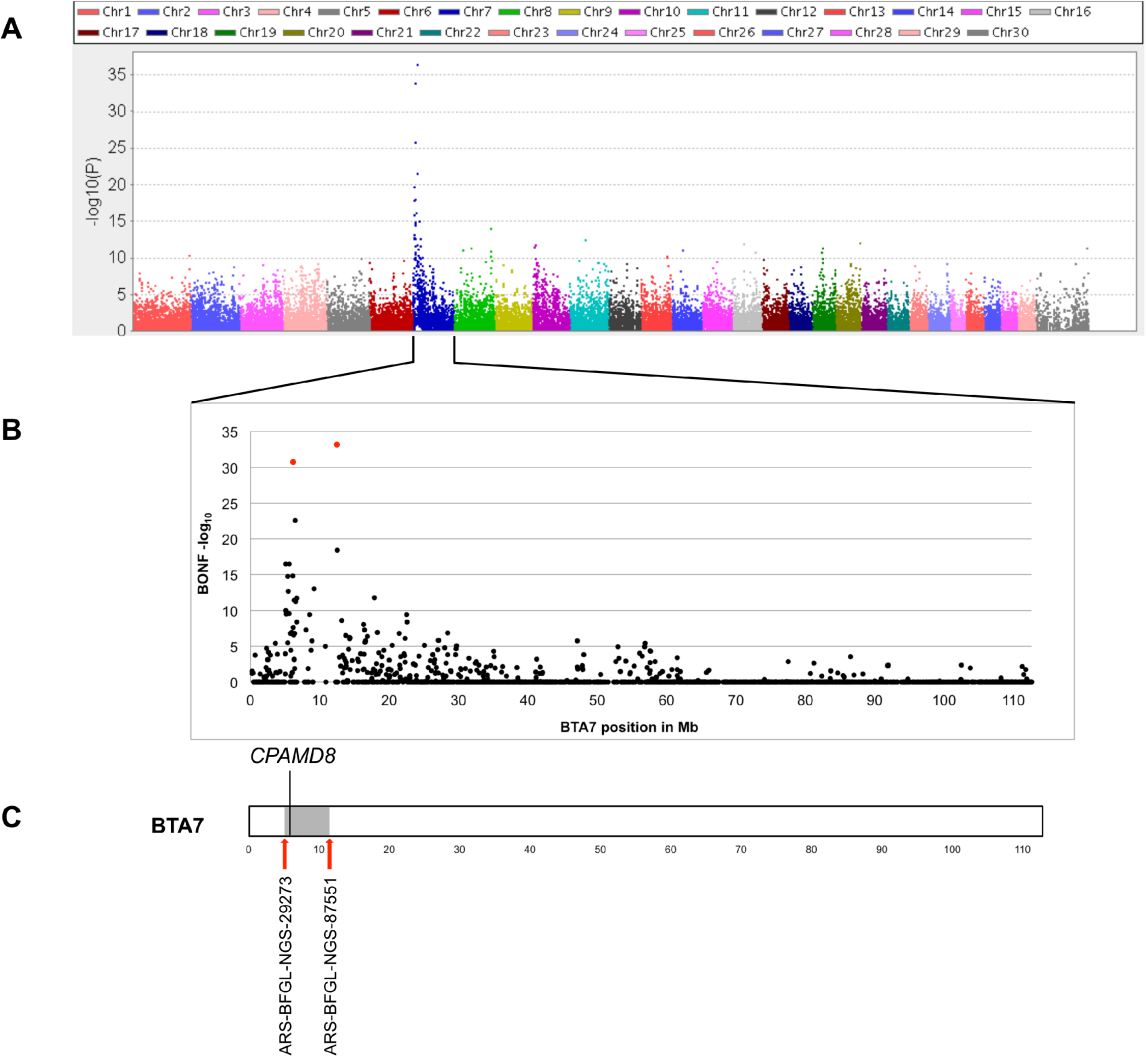
Results of genome-wide association study (GWAS) using SNP data of 26 cases and 88 Holstein control animals. (A) Manhattan plot (generated using Haploview [23]) and calculated -log_10_ p values showing an association with the cataract phenotype on BTA7, (B) Detailed view of association on BTA7 with -log_10_ Bonferroni-adjusted p values. Red symbols showing associations of 5.44x10^-30^ at position 6,166,179 and 1.85x10^-32^ at position 12,429,691, (C) Region of extended homozygosity on BTA7 in cataract cases (grey symbol). *CPAMD8* (5,995,747 to 6,095,877) is located at the proximal end of the 4.7 Mb region of extended homozygosity from 5,639,104 to 10,406,009.

Case-control analysis of 26 cases and 88 controls revealed an association with the cataract phenotype on bovine chromosome 7 (BTA7) (p values: 4.41x10^-37^ (Bonferroni–adjusted p value: 1.85x10^-32^) at position 12,429,691 and 1.30x10^-34^ (Bonferroni–adjusted p value: 5.44x10^-30^) at position 6,166,179) (all positions refer to assembly UMD_3.1) (Fig 3). Regarding the proposed autosomal recessive inheritance, we searched for regions of extended homozygosity in cases of congenital cataracts. A 4.7 Mb interval from 5,639,104 to 10,406,009 on BTA7 (UMD_3.1) was detected harboring 76 SNPs. Due to the positional overlap of the association on BTA7 and the region of homozygosity on BTA7, we searched for positional and functional candidate genes within this region. However, none of the 166 annotated genes located in the region of extended homozygosity had so far been associated with congenital cataract.

### Whole genome re-sequencing and identification of a variant in the *CPAMD8* gene associated with congenital cataract formation in cattle

Due to the lack of functional candidate genes in the associated region on BTA7, a whole genome re-sequencing was performed. Based on the pedigree analysis two trios were sequenced. The first trio consisted of one case (#489, Fig 1D) and its parents (#870 and #2104). The second trio included the grandmother (#501) and a cross between the grandmother #501 and the father #870 of this case #489. 2019 variants in the homozygous state were called for the affected animal in the associated interval from 4 to 13 Mb on BTA7. The detected variants were filtered for their predicted effect on the amino acid sequence. Seven variants with non-synonymous coding and one variant leading to a premature stop codon remained after filtering. Six of seven variants with non-synonymous coding affected olfactory receptors (olfactory receptor 10H1-like, 10H1 and 10H4) and were excluded from further analysis due to their biological function. Remaining variants in *GTPBP3* (GTP binding protein 3, g.5711730T>C) and *CPAMD8* (C3 and PZP like, alpha-2-macroglobulin domain containing 8, g.5995966C>T; positions refer to accession number AC_000164.1, UMD_3.1) were validated by PCR and subsequent melting curve analysis using FRET technology. The only variant that remained perfectly associated with the cataract phenotype was the chain termination mutation detected in *CPAMD8* (g.5995966C>T). *CPAMD8* is located from 5,995,747 to 6,095,877 on BTA7 (UMD_3.1, AC_000164.1) and contains 42 exons. The gene is located at the proximal end of the detected region of extended homozygosity on BTA7 and at a distance of around 70 kb from a SNP at position 6,166,179 (Bonferroni-adjusted p value of 5.44x10^-30^, UMD_3.1, AC_000164.1), which was identified previously by GWAS. The identified variant g.5995966C>T was located within the first exon of the *CPAMD8* gene at position c.220 (UMD_3.1) and has not yet been described. The nonsense mutation p.Gln74* results in a null allele. Homozygous mutant cases therefore lack CPAMD8 protein.

### Genotyping of variant g.5995966C>T

Genotyping of 1,248 animals was performed to verify the variant g.5995966C>T in *CPAMD8* (Table 1). All animals with cataract phenotype (n = 31) were tested T/T. In total 161 cattle were genotyped C/T, including the parents of affected cattle. 1,055 randomly tested control animals were tested homozygous C/C. All pedigrees of heterozygous animals showed an inheritance with the predicted founder, sire #780.

**Table 1:** Detection of *CPAMD8* g.5995966C>T in different cattle breeds.

Genotype	No. of cattle
**T/T**	32^a)^
**C/T**	161^b)^
**C/C**	1,055^c)^
**total**	1,248

a) The 32 T/T individuals include 31 cases and one HF animal with unknown phenotype (#183). Both parents of #183 were tested heterozygous C/T and were related to the predicted founder sire #780.

b) All heterozygous tested HF cattle were related to sire #780.

c) The 1,055 C/C individuals include 1,046 HF and nine animals of other breeds, i.e. Angus, Charolais, Jersey, Limousin, White Galloway.

Besides the 31 tested cataract cases, there was an additional animal (#183) genotyped T/T. Unfortunately, the phenotype of the animal was unknown, since it had already been culled by the time of genotyping. However, the father (#554) and the maternal grandfather (#870) of #183 were tested heterozygous C/T and were related to sire #780.

### RNA expression of bovine *CPAMD8*

RNA was extracted from the lenses of three animals, one with cataract (mature to hypermature cataract, #489), one adult control and one fetal control animal. Furthermore, lung and thyroid gland tissues from two animals, one with cataract (#489) and one adult control animal, were used as control samples (Table 2). Other ocular tissue samples were not considered. Except for cataracts, observed ocular anomalies were not congenital. Anomalies as listed in S1 Table, developed due to ongoing lens dissolution and subsequent inflammatory processes affecting the eyes of cataract cases. Due to the physiological condition of the other ocular structures, these tissues were not considered for RNA analyses. Although RNA from cataractous and adult lens extracts showed low RNA quality (RIN factors <6), *CPAMD8* RNA was detected in all tested tissue samples. However, an exact quantification relative to the reference gene was not possible. Despite the low quality, *CPAMD8* RNA was detectable in lens materials of cataractous, fetal and healthy adult lenses.

**Table 2:** Tissue samples for RNA analyses of *CPAMD8*, including ddPCR results and RNA integrity number (RIN) from RNA quality measurement using RNA 6000 Pico Kit (Agilent Technologies, Waldbronn, Germany).

Tissue samples	RIN	Mean copy numbers *CPAMD8* (CV^1^)	Mean copy numbers *GAPDH* (CV^1^)
**Lung-healthy control**	7.4	23.3 (0.107)	99 (0.0808)
**Thyroid gland-healthy control**	6.9	11.5 (0.260)	1442.5 (0.0947)
**Lung case**	8.3	59.5 (0.032)	127 (0.0394)
**Thyroid gland case**	7.4	8.2 (0.012)	228 (0.0263)
**Adult healthy lens**	2.4	0.125 (0.360)	120.5 (0.0622)
**Fetal lens**	7.1	0.665 (0.0226)	597.5 (0.0410)
**Cataractous lens**	1	0.023 (0.231)	0.32 (0.0625)

1) CV = Coefficient of variation

Annotation of *CPAMD8* is based on assembly UMD_3.1, AC_000164.1. Expression of corresponding isoforms was tested by PCR for lens tissue. There was no indication of expression of alternative isoforms as e.g. ENSBTAP00000036263, annotated in Ensembl database.

### Immunohistochemical detection of CPAMD8

**Fig 4:**
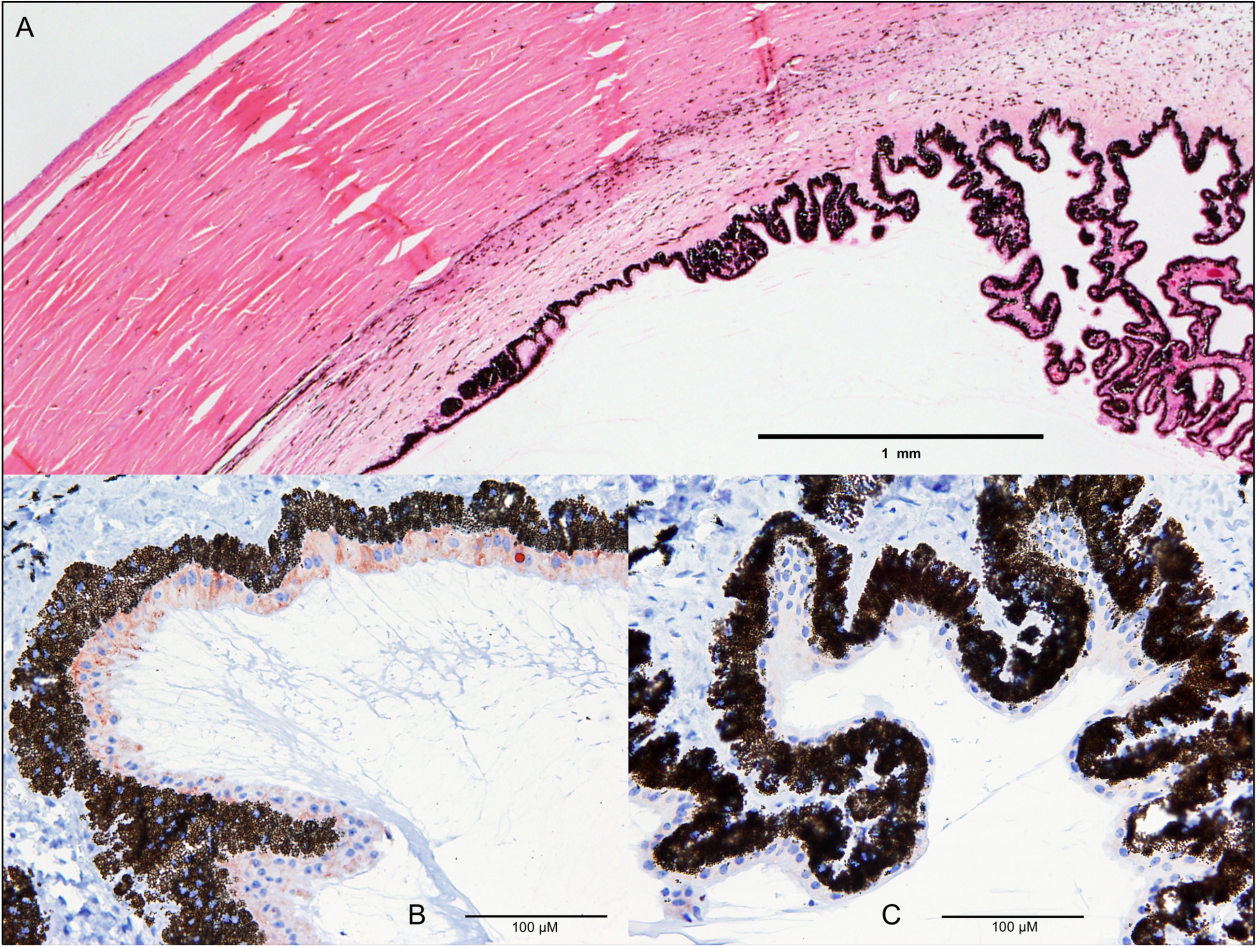
Loss of CPAMD8 expression in cataract cases. Overview of cornea and ciliary body (A, H&E staining). Physiologically, CPAMD8 is detectable by immunohistochemical staining in the epithelium of the ciliary body as cytoplasmic and vesicular staining in light brown (adult control sample) (B). In the cataract ciliary body epithelium, no CPAMD8 immunoreaction is detectable (C); CPAMD8 antibody; visualization by AEC.

In healthy fetal and adult control cattle, CPAMD8 was detectable by immunohistochemistry in the epithelium of the ciliary body, where the staining was related to intracytoplasmic organelles (Fig 4B). In contrast, in the ciliary body epithelium of cataract cases, no CPAMD8 immunoreaction was detectable (Fig 4C). In cataract and control cases alike the retina, the epithelium of the lens and the lentil fibers showed no antibody reaction for CPAMD8 in the immunohistochemical staining. However, using Western blotting a faint band at the expected size of CPAMD8 was detectable in normal fetal and normal adult but not adult cataractous lenses (S1 Fig).

## Discussion

In the present study we identified a chain termination mutation (g.5995966C>T) in the first exon of *CPAMD8* associated with congenital cataract formation in Red Holstein Friesian cattle. Information about the potential biological function of CPAMD8 is relatively scarce. Recently, Cheong et al. (2016) identified several mutations (missense, splice-site and frameshift variants) in *CPAMD8* associated with ASD in humans, a disorder causing malformations of the anterior segment of the eye [15]. Affected patients showed several iridal malformations and cataracts, mainly of the posterior cortical type [15]. Observed bovine cataract cases with congenital mature cataracts did not show any other congenital anomalies of the iris or other ocular tissues. Likewise identified mutations in ASD patients and cataract cattle observed here differed regarding location and effects on the protein. Cheong et al. (2016) identified a homozygous missense mutation (c.4351T>C, p.S1451P) in exon 32 in one family affecting the conserved A2M domain of the CPAMD8 protein. In two other families two compound heterozygous mutations were identified respectively, causing aberrant pre-mRNA splicing resulting in an in frame deletion/insertion or smaller transcripts. The identified mutation c.2352_2353insC in family 2 is predicted to cause a premature termination in exon 18 of 42 resulting in a truncated protein [15]. Here a missense mutation (g.5995966C>T) in exon 1 of bovine *CPAMD8* gene is reported leading to a premature termination that is predicted to cause a loss of protein in homozygous affected animals. In contrast, findings of Cheong et al. (2016) showed that a truncation of the protein by missense mutation, compound heterozygous frameshift mutations or aberrant pre-mRNA splicing are causing malformations of the anterior segment of the eye including cataracts.

Furthermore, we detected CPAMD8 in the ciliary body epithelium of healthy fetal and adult cattle, whereas no expression was detected in cataract cases. The non-pigmented part of the ciliary body secretes the aqueous humor by a complex process including steps of diffusion and ultrafiltration [24, 25]. Proteins in aqueous humor, although the concentration is rather low [25], are thought to derive from several sources like the plasma and the ciliary body itself [26, 27]. Goel et al. (2013) identified 2,815 proteins in the ciliary body, 896 of which are unique to the tissue and cannot be found in the plasma [27]. Due to our observation of CPAMD8 in the non-pigmented epithelium of the ciliary body of healthy cattle, we assume that the CPAMD8 protein is usually secreted into the aqueous humor in the physiological state. This is supported by previous reports of CPAMD8 protein in human ciliary body [27] and aqueous humor [28–30]. To the best of our knowledge there have been no reports about defects of any other ciliary body proteins causing cataracts so far. CPAMD8 was not detectable by immunohistochemistry in lens tissues of fetal (approximately 11 weeks of age) and adult controls as well as cataract animals. However, the presumed absence of CPAMD8 in fetal and normal adult lenses was most likely only due to the reduced intensity of the immunohistochemical staining. Because, when using Western blotting as a more robust and quantitative method [31], a faint band at the expected size of CPAMD8 was also detectable in normal fetal and adult but not cataractous lenses (S1 Fig). So far there have been no reports of CPAMD8 in mammalian lens tissues, although it was detected in other ocular tissues and fluids e.g. aqueous humor [28–30], ciliary body [27], zonular fibres [32], vitreous humor [33], iris [34, 35], and choroid-retinal pigment epithelial complex [36].

We investigated *CPAMD8* RNA expression in several bovine tissues including adult, fetal and cataractous lenses. Likewise, Cheong et al. (2016) reported an expression of *CPAMD8* in human fetal lens in increasing levels from week 9 to 22 of gestation [15], at a time when the first lens fiber cells are already being formed (by the seventh week of gestation) [37]. Due to the similar pregnancy periods of humans and cattle, this clearly corresponds with our findings of *CPAMD8* expression in bovine fetal lens tissue derived from a fetus of approximately 10 weeks of age according to the measured crown-rump length. Furthermore, *CPAMD8* expression was detected in adult and cataractous lens material, indicating a postpartal expression of *CPAMD8*. Due to degradation in cataractous lenses RNA quantification was not possible.

Nevertheless, our findings of *CPAMD8* RNA expression in lens tissues and CPAMD8 protein in ciliary body and lenses of healthy cattle are in accordance with previous reports [15, 27]. In which way lens development might be influenced by the loss of CPAMD8 protein is a question that requires further investigation.

Concerning the structure of CPAMD8, the protein belongs to the C3/Α2M family, which has a further six members, including A2M, PZP, CD109 and the complement components C3, C4, C5 [16]. The *CPAMD8* gene has some structural similarities in common with other family members like, for example, A2M-associated domains and a Kazal-type serine protease inhibitor/follistatin-like domain, which is highly conserved across vertebrate species [16].

Functionally, members of the C3/A2M family are part of the innate immune system [17, 18]. Complement components recognize and eliminate pathogens, i.e. elimination is achieved by opsonization, lysis of pathogens and inflammatory response [18]. Alpha-2-macroglobulin (Α2M) proteins are able to capture proteases of endogenous and exogenous origin and are evolutionarily highly conserved elements [17]. Ji et al. (2016) identified proteomic changes of the complement components C3 and C5 in aqueous humor of cataract patients and controls, being associated with cataract development.

Seeking a potential role for CPAMD8 during cataract development, structurally similar Α2M was identified acting as a carrier of specific growth factors, like as e.g. TGF*β* [38]. TGF*β* family members are present in ocular surroundings, i.e. lens [39, 40], aqueous and vitreous humor [30, 33, 41]. Changes in TGF*β* levels are known to result in cataract development [42], indicating a strong regulation of the expression and bioavailability of TGF*β*. Also, TGF*β* signaling is thought to have a key role during terminal fiber cell differentiation [43]. It has already been shown that Α2M *in vitro*, when present in ocular media, blocks cataractous changes induced by TGF*ß* [44], suggesting that suitable levels of molecules like A2M in the ocular media have a protective function for lens cells against the damaging effects of TGF*ß*. Here, reduced levels of A2M or similar molecules may predispose the lens to cataractogenesis. However, cataractous changes induced by TGF*ß* are usually similar to those found in anterior subcapsular cataracts [45]. ASD patients with mutations in *CPAMD8* mainly suffer from cortical [15] and affected cattle from complete mature cataracts. These differences in cataract manifestation may indicate that CPAMD8 and Α2M, in terms of cataract prevention, act by different pathophysiological mechanisms, although they have structural similarities and have expression in aqueous humor and ciliary body in common.

Clinical examination of affected cattle in this study revealed a mature cataract at time of birth. With age, the cataract manifestation changed from mature to hypermature. Histological results, such as Morgagnian globules, liquefaction, the absence of lens epithelium and reduction of lens size, are typical for hypermature cataract progression of cortical cataracts [46]. Alterations leading to congenital, mature cataracts most likely affect a stage in embryonic lens development when lens fiber cells are already formed. Affected cattle seemed to have normal-sized lenses at the time of birth, which supports this hypothesis.

Besides the inherited form of cataractogenesis, intrauterine infections play a crucial role in cataract development. Bovine Viral Diarrhea-Mucosal Disease (BVD-MD) is known to be responsible for the development of cataracts in cattle [47]. Twenty-two of the 31 sampled cataract cases were tested for BVD-MD by antigen-ELISA test with negative results. Therefore, an intrauterine infection as the cause of the cataracts could be excluded.

Our findings are not only interesting from a scientific point of view, they also have substantial relevance for practical breeding. Genotyping of male offspring of identified carriers revealed further heterozygous sires. Two of these tested heterozygous males were ranked top ten in the active, daughter-proven, artificially inseminated German Red Holstein population (ranked by relative breeding value) in August 2015 [48].

In summary, we have discovered a nonsense mutation (g.5995966C>T, p.Gln74*) in the bovine *CPAMD8* gene which coincides perfectly with cataract in the tested animals. We demonstrated that CPAMD8 is expressed in the ciliary body epithelium and lens of the unaffected eye and assume that a secretion of CPAMD8 from the epithelium into aqueous humor takes place under normal circumstances. The question of how in detail the loss of CPAMD8 in these cases results in cataract development needs to be examined in further studies. Nevertheless, our data provide convincing evidence that the absence of CPAMD8 leads to congenital cataract formation during embryonic development in Red Holstein Friesian cattle and are in accordance with previous findings that CPAMD8 is involved in eye development in humans [15].

## Materials and methods

### Animal ethics/ethics statement

The current study was performed in accordance with the ethical guidelines of the University of Goettingen and the German Animal Welfare act. Genotyping data and blood sampling had been done independent of the project in the frame of routine diagnostic parentage control and genomic selection programs of the German Holstein Friesian Association (DHV). The data sets were provided by DHV with written owner consent. Collection of blood samples within the breeding programs have been conducted exclusively by state-licensed veterinarians in accordance with the German Animal Welfare Act (§6 Abs. 1 Satz 2 TierSchG). Tissue samples and other native materials were obtained during or after regular slaughtering processes at an abattoir according to §4 of the German Animal Welfare Act. Therefore no formal ethical approval was required, since no other samples were collected for this study.

### Animal material and sample collection

DNA samples from 31 affected (24 female, 7 male) Holstein Friesian cattle were available. Genomic DNA had been extracted from EDTA blood using a modified salting out procedure [49] or MagNa Pure LC DNA Isolation Kit I (Roche Diagnostics, Mannheim, Germany). DNA of related sires and other animals in the study was extracted from semen using a modified salting out method or obtained from the DNA depository of the Institute of Veterinary Medicine (Goettingen). Sample material for whole genome re-sequencing was extracted from EDTA blood as described above or semen using a phenol chloroform extraction method [50]. Lenses were obtained during regular slaughtering and stored at -80°C or fixed in 10% neutral buffered formalin for histological analysis. Fetal lenses were prepared from fetus (approximately 79 (immunohistochemistry) and 69 (RNA analysis) days of age according to measured crown-rump length) of dam during regular slaughtering and handled as described above.

### Genome-wide association study (GWAS) and homozygosity mapping

Genotyping was performed using the BovineSNP50 BeadChip (Illumina, San Diego, USA) according to the manufacturer´s protocols. In total, 26 affected and 88 control HF cattle were genotyped. Final reports were generated using GenomeStudio V2011.1 (Illumina, San Diego, USA). Statistical analyses were performed using PLINK software [51]. Genotype data were filtered for quality control criteria as SNP call rate >95% and minor allele frequency ≥ 5%, reducing the final data set to 46,075 SNPs.

Firstly, a case-control association was calculated. P values were Bonferroni-adjusted to correct for multiple testing. Regions of extended homozygosity with shared alleles in cases were analyzed in a second step. Regions larger than 1 Mb were included in the analysis. Genomic positions refer to assembly UMD_3.1.

### Genome re-sequencing and variant calling

Whole genome shotgun sequencing was conducted on five cattle: one affected animal (#489) and its unaffected parents (#870 and #2104), one unaffected maternal grandmother (#501) and an unaffected cross between the maternal grandmother (#501) and the father of the affected cattle (#870). Sequences were deposited with the European Nucleotide Archive (ENA) under accession number PRJEB20549 (http://www.ebi.uk/ena/data/view/PRJEB20549).

Sequencing libraries were prepared from 100ng DNA using the NEBNext Ultra DNA Library Prep Kit for Illumina (New England Biolabs, Ipswich, USA) according to the manufacturer’s instructions. Paired-end sequencing was conducted on a NextSeq500 (Illumina, San Diego, USA).

An average of 85M (SD: 7.8M) reads were generated per animal, of which 83% (SD: 1.3%) were mapped to the bovine reference genome (UMD_3.1) resulting in a mean coverage of 7.8-fold (SD: 0.78).

Sequences were mapped using a BWA software package [52]. Variants were named using GATK [53]. Effects on the amino acid sequence were predicted by the SNPeff tool [54].

### Genotyping by PCR using FRET technology

SNP genotyping was done by fluorescence resonance energy transfer-PCR [55]. Primers were designed using NCBI primer-BLAST [56] with the following sequences: CPAMD8_FRET_93bp_F: GTTCTTGCTGCTGCTGCTG and CPAMD8_FRET_93bp_R: CAGCCAGCCTTCTCTCGC. Probes, bCPAMD8_probe: Cy5-GCGCTGAGCAGCCCCA-PHO and bCPAMD8_anchor: CGCCGCGGGACGGCG-Flc, were designed using the MeltCalc software [57, 58]. Primers and probes were synthesized by Sigma-Aldrich (Taufkirchen, Germany).

PCR was performed on a LightCycler480 or LightCycler480 II (Roche Diagnostics, Mannheim, Germany) in a total volume of 25μl using FastStart *Taq* DNA Polymerase, dNTPack (Roche Diagnostics, Mannheim, Germany). One reaction mix included 1.5U Faststart *Taq* DNA Polymerase, 200μM dNTP, 10μM of each primer and probe, 1x GC-RICH solution, 4.5mM MgCl_2_, 1x PCR reaction buffer (including 20mM MgCl_2_), and 60-150ng of DNA. Cycling conditions were 95°C for 10 min, followed by 35 to 40 cycles of 95°C for 10 sec, 59°C for 20 sec and 72°C for 10 sec. Final elongation step was 72°C for 5 min. Melting was done using an appropriate filter set and the following program: 95°C for 30 sec, 45°C for 30 sec, 75°C continuous acquisition mode (2 to 3/°C), ramp rate 0,29°C/sec, followed by 45°C for 30 sec.

### RNA extraction and cDNA product amplification

RNA was extracted from 50 to 100 mg tissue material homogenized in 900 μl TRIzol® (Thermo Fisher Scientific) using Magna Lyzer Green Beads (Roche Diagnostics, Mannheim, Germany). After homogenization, RNeasy Plus Universal Kit (Qiagen, Hilden, Germany) was used following the manufacturer´s protocol. RNA quality control was tested using Agilent RNA 6000 Pico Kit (Agilent Technologies, Waldbronn, Germany). cDNA synthesis was performed using Maxima H Minus First Strand cDNA Synthesis Kit, with dsDNase (Life Technologies). Oligo(dt) and Random Hexamer primers were used for reverse transcription.

### Digital droplet PCR assay for RNA expression analysis

*CPAMD8* RNA expression analyses were performed on a QX200Droplet Digital PCR system (Bio-Rad Laboratories, Munich, Germany) using RNA samples of one cataract case, one healthy adult and one fetal control. One technical replication was performed for every sample. Primers were designed using NCBI primer-BLAST [56]. Sequences of the primers were: CPAMD8_F: GATGGGAAGTCCGTCAGACC and CPAMD8_R: TGGACTCTCTCGTTGGGACA. As controls served *ACTH* and *GAPDH* with the following primer sequences: ACTB_cDNA_F: ACAGGATGCAGAAAGAGATCAATG, ACTB_cDNA_R: GTACTCCTGCTTGCTGATCCAC, GAPDH_cDNA_F: GTATGATTCCACCCACGGCA and GAPDH_cDNA_F: ACCACATACTCAGCACCAGC. All primers were synthesized by Sigma-Aldrich (Taufkirchen, Germany).

PCR mix at a total volume of 20μl included 10μl of QX200™ ddPCR™ EvaGreen Supermix (Bio-Rad Laboratories, Munich, Germany) and 2 μM of each primer. 1 μl to 2 μl of diluted samples (*CPAMD8* 1:2, *ACTH* and *GAPDH* 1:250) were used in PCR. cDNA of cataract case was used without dilution (2μl). Droplets were generated using QX200 Droplet Generator (Bio-Rad Laboratories, Munich, Germany). PCR was performed in a thermocycler (Biometra, Göttingen, Germany) and cycling conditions were as follows: 95°C for 10 min, followed by 45 cycles of 95°C for 30 sec and 60°C for 1 min. Final droplet stabilization was performed at 98°C for 10 min. Droplets were analyzed using the QX200 Droplet Reader (Bio-Rad Laboratories, Munich, Germany).

### Histology

Tissues were fixed in 10% neutral buffered formalin for at least 48 h, embedded in paraffin, and 1–3μm tissue sections were cut with a microtome. Sections were mounted on glass slides, dried and stored for histological and immunohistochemical staining procedures. The morphological evaluation was performed on hematoxylin and eosin (H&E) stained sections.

### Immunohistochemistry and Western blotting

For protein analyses four commercially available antibodies were used (S2 Table). Specificity and sensitivity of the antibodies were tested using different bovine tissues and an *in vitro* expressed recombinant bovine CPAMD8 epitope (S1 Appendix, S2 Fig). Antibody concentrations were titrated until an optimal signal to noise ratio was obtained. For immunohistochemistry antibodies HPA031327 and HPA031328 were initially tested and HPA031328 gave best signals. Therefore, this antibody was used for the further studies. For immunohistochemistry paraffin-embedded tissue sections were deparaffinized and rinsed with TBS. For antigen epitope retrival, slides were heated in a microwave at 200W for 5 x 3 min in TE-buffer (10mM Tris, 1mM ETDA, pH 9.0) and unspecific antibody binding was blocked with 3% hydrogenperoxide and 10% donkey serum in TBS-Triton (50mM Tris, 150mM NaCl, 0.1% Triton X-100).

Immunoreaction was performed by incubation with the affinity purified polyclonal rabbit anti-CPAMD8 antibody (HPA031328, Sigma-Aldrich) (1:50, according to manufacturer’s instructions) diluted in TBS-Triton followed by the secondary donkey-derived anti-rabbit antibody (RPN1004, GE Healthcare, Hamburg, Germany) (1:100) for 2 h and 1 h, respectively. After each step, slides were rinsed thoroughly with TBS-Triton. The immunoreaction was visualized using Avidin-conjugated peroxidase as enhancer and AEC (3-Amino-9-ethylcarbazole) as chromogen. Sections were lightly counterstained with hematoxylin.

For Western blotting proteins were lysed using lysis buffer containing 50mM Tris, 150mM NaCl, 1mM EDTA, 1mM EGTA, 5% glycerol, 1% Triton X-100, 0.1% DTT and EDTA free protease inhibitor cocktail (Roche Diagnostics, Mannheim, Germany). Homogenization of samples was done using DIAX900 Homogenizer (Heidolph, Schwabach, Germany) or MagNA Lyser Green Beads (Roche Diagnostics, Mannheim, Germany) followed by an incubation for 1 hour and centrifugation for 10 min at 500g and 4°C. Proteins were quantified by Bradford method.

60μg of proteins were analyzed using SDS-Page (4–12% Bis Tris- Plus gels or 8% Bis Tris Plus gels, Life Technologies). As positive controls human CPAMD8 over-expression Lysate (OriGene Technologies, Rockville, USA) and 5μl of *in vitro* produced bovine CPAMD8 were used. Proteins were transferred to nitrocellulose membrane using semi-dry blotter (Brenzel Bioanalytik, Lahntal, Germany). After transfer membranes were blocked with 5% non-fat dry milk in TBS-T for 2 h, followed by incubation with anti-CPAMD8 antibody (S2 Table, dilution 1:200) for 1 h at room temperature. Secondary goat anti-rabbit IgG (H + L)-HRP conjugate antibody (Bio-Rad Laboratories, Munich, Germany) was diluted 1:5000 and also incubated for 1 h at room temperature. Detection of chemiluminescent signals was performed using Amersham ECL detection reagent (GE Healthcare, Hamburg, Germany) and membranes were exposed to X-ray films (GE Healthcare, Hamburg, Germany).

## Supporting information

S1 Fig Western blot analysis of normal adult, normal fetal and adult cataractous lenses. Protein extracts of a normal adult lens (lane 2), normal fetal lens (lane 3) and adult cataractous lens (lane 4) were loaded on a 4–12% gradient SDS-PAGE. As positive control human CPAMD8 over-expression lysate was used (lane 1). Proteins were transferred to nitrocellulose membranes and filters were blocked with 5% non-fat dry milk in TBS-T followed by incubation with anti-CPAMD8 antibody HPA031328 (S2 Table, dilution 1:200). Detection of chemiluminescent signals was performed using Amersham ECL detection reagent and membranes were exposed to X-ray films. CPAMD8 bands in lane 1 (human control lysate), 2 (bovine normal adult lens) and 3 (bovine normal fetal lens) are indicated with an arrow. Protein sizes are indicated in kDa. Scans of X-ray films were cropped using GIMP 2.8.18 (GNU Image Manipulation Program).
(TIFF)

S2 Fig Analysis of antibody specificity and sensitivity using an *in vitro* expressed bovine CPAMD8 epitope and CPAMD8 antibody HPA031328. Five μl of *in vitro* produced bovine CPAMD8 epitopes were loaded on a 4–12% gradient SDS-PAGE (lane 1, lane 2). Proteins were transferred to nitrocellulose membranes and filters were blocked with 5% non-fat dry milk in TBS-T followed by incubation with anti-CPAMD8 antibody HPA031328 (S2 Table, dilution 1:200). Detection of chemiluminescent signals was performed using Amersham ECL detection reagent and membranes were exposed to X-ray films. The band in lane 1 shows the expected size of the epitope (approx. 33 kDa) and is indicated with an arrow. In lane 2 another *in vitro* expressed epitope lysate was loaded showing no reactivity. Lane 3 shows a negative control lysate. Protein sizes are indicated in kDa. Scans of X-ray films were cropped using GIMP 2.8.18 (GNU Image Manipulation Program).
(TIFF)

S1 Table Summary of the clinical findings of cataract cases. Consciousness, posture, gait, swallowing and tongue tone were normal in all cases. Behavior differed from calm alertness to anxiety. If not stated otherwise the ocular fundus was without pathological findings.
(DOCX)

S2 Table Anti-CPAMD8 antibodies used for Western blotting and immunohistochemistry. 1) Sigma-Aldrich Chemie (Taufkirchen, Germany); 2) OriGene Technologies (Rockville, USA).
(DOCX)

S1 Appendix Materials and methods used for *in vitro* expression of a bovine CPAMD8 epitope. (DOCX)
