# Supplementary figures and images for: Morgagnian cataract resulting from a naturally occurring nonsense mutation elucidates a role of CPAMD8 in mammalian lens development
Source: PLoS One. 2017 Jul 6;12(7):e0180665. doi: 10.1371/journal.pone.0180665 (PMC5500361; doi:10.1371/journal.pone.0180665)

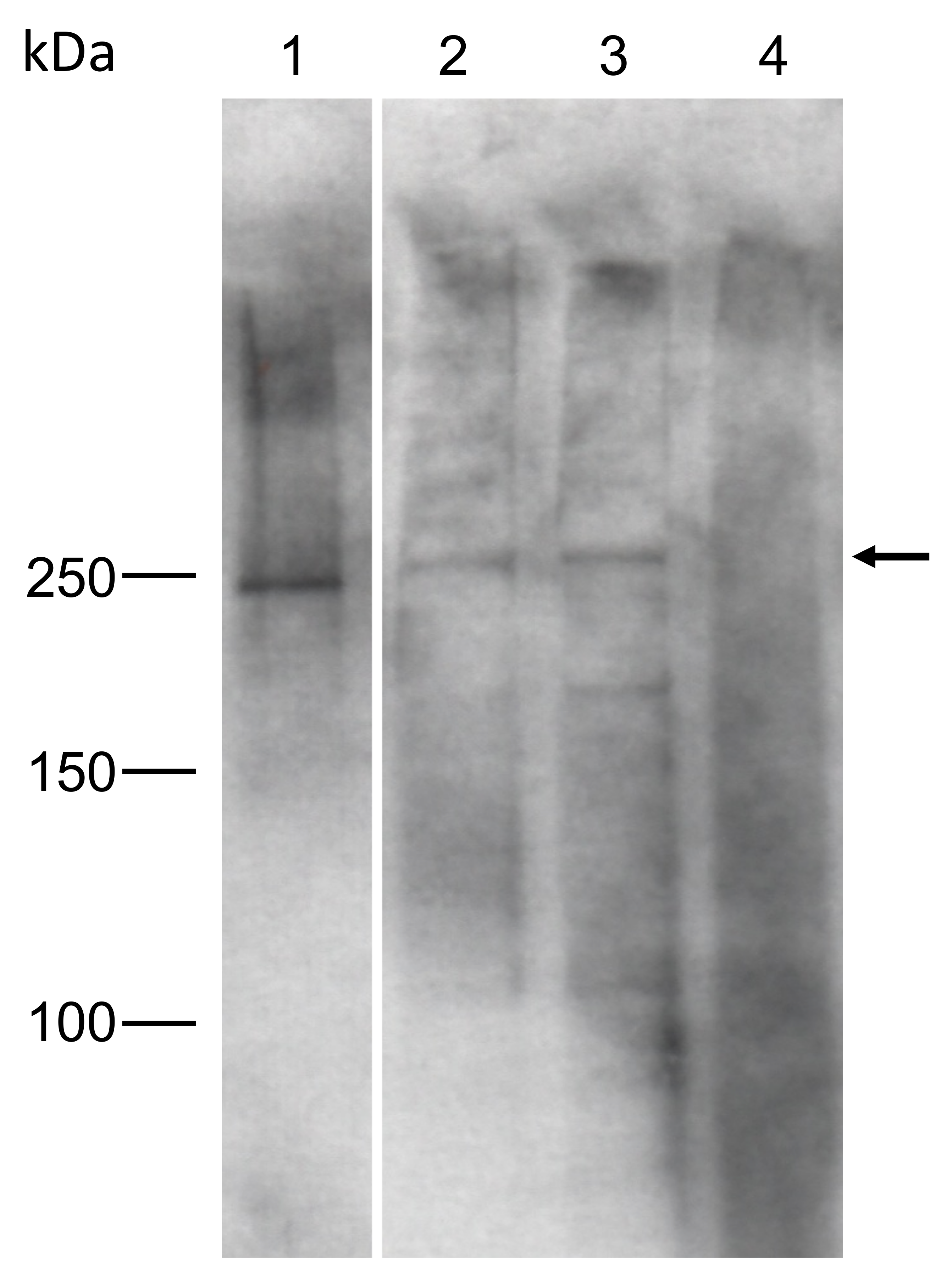

Supplement: S1 Fig — Protein extracts of a normal adult lens (lane 2), normal fetal lens (lane 3) and adult cataractous lens (lane 4) were loaded on a 4–12% gradient SDS-PAGE. As positive control human CPAMD8 over-expression lysate was used (lane 1). Proteins were transferred to nitrocellulose membranes and filters were blocked with 5% non-fat dry milk in TBS-T followed by incubation with anti-CPAMD8 antibody HPA031328 (S2 Table, dilution 1:200). Detection of chemiluminescent signals was performed using Amersham ECL detection reagent and membranes were exposed to X-ray films. CPAMD8 bands in lane 1 (human control lysate), 2 (bovine normal adult lens) and 3 (bovine normal fetal lens) are indicated with an arrow. Protein sizes are indicated in kDa. Scans of X-ray films were cropped using GIMP 2.8.18 (GNU Image Manipulation Program). (TIFF) [file pone.0180665.s001.tiff]

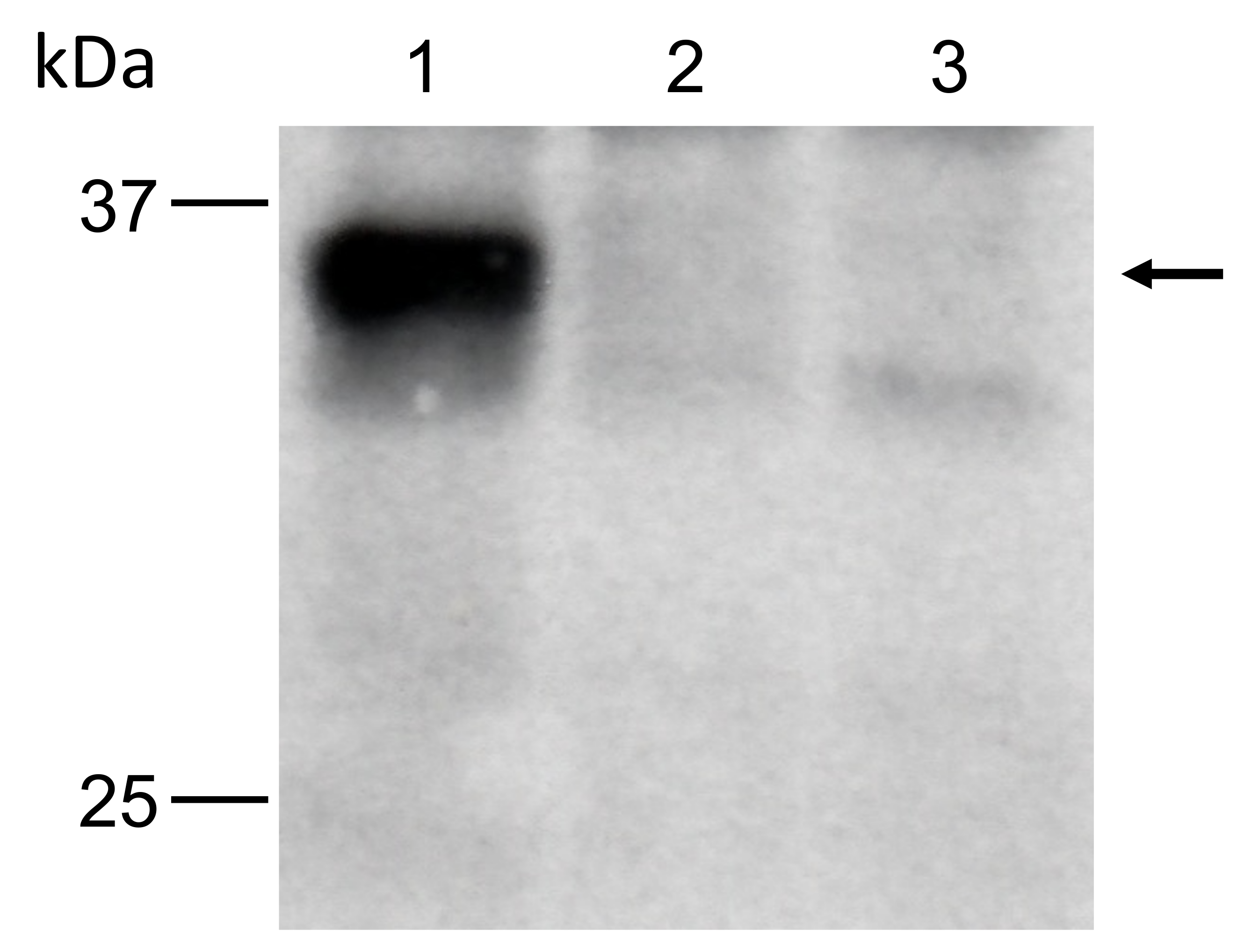

Supplement: S2 Fig — Five μl of in vitro produced bovine CPAMD8 epitopes were loaded on a 4–12% gradient SDS-PAGE (lane 1, lane 2). Proteins were transferred to nitrocellulose membranes and filters were blocked with 5% non-fat dry milk in TBS-T followed by incubation with anti-CPAMD8 antibody HPA031328 (S2 Table, dilution 1:200). Detection of chemiluminescent signals was performed using Amersham ECL detection reagent and membranes were exposed to X-ray films. The band in lane 1 shows the expected size of the epitope (approx. 33 kDa) and is indicated with an arrow. In lane 2 another in vitro expressed epitope lysate was loaded showing no reactivity. Lane 3 shows a negative control lysate. Protein sizes are indicated in kDa. Scans of X-ray films were cropped using GIMP 2.8.18 (GNU Image Manipulation Program). (TIFF) [file pone.0180665.s002.tiff]
