# Supplementary material for: Morgagnian cataract resulting from a naturally occurring nonsense mutation elucidates a role of CPAMD8 in mammalian lens development
Source: PLoS One. 2017 Jul 6;12(7):e0180665. doi: 10.1371/journal.pone.0180665 (PMC5500361; doi:10.1371/journal.pone.0180665)
Supplement: S1 Table — Consciousness, posture, gait, swallowing and tongue tone were normal in all cases. Behavior differed from calm alertness to anxiety. If not stated otherwise the ocular fundus was without pathological findings. (DOCX) [file pone.0180665.s003.docx]

| **Cattle no.** | **Age in months** | **Clinical findings** | |
| --- | --- | --- | --- |
|  |  | Left eye | Right eye |
| 224 | 13 | - partial blindness - inducible nystagmus - mydriasis - cataract - microphakia - irregular shaped lens - posterior synechia | - complete blindness - no palpebral reflex - mydriasis - cataractous lens disconnected from iris and ventrally subsided - glaucoma - uveitis - retinitis - exophthalmos |
| 227 | 13 | - partial blindness - low-grade ventral strabismus - palpebral reflex slightly reduced - sensibility around the eye slightly reduced - mydriasis - cataract - mikrophakia - irregular shaped lens continuing into the anterior chamber - posterior synechia | - partial blindness - mydriasis - cataract - mikrophakia |
| 489 | 31.5 | - partial blindness - mydriasis - cataract - mikrophakia | - partial blindness - mydriasis - cataract - mikrophakia - exophthalmus |
| 908 | 30 | - partial blindness - mydriasis - cataract - irregular shaped lens with attached fibrils continuing into posterior chamber - mikrophakia - cloudy and filamentous content in posterior chamber | - partial blindness - mydriasis - cataract - mikrophakia - irregular shaped lens located lateral continuing into anterior chamber |
