# Supplementary material for: Morgagnian cataract resulting from a naturally occurring nonsense mutation elucidates a role of CPAMD8 in mammalian lens development
Source: PLoS One. 2017 Jul 6;12(7):e0180665. doi: 10.1371/journal.pone.0180665 (PMC5500361; doi:10.1371/journal.pone.0180665)
Supplement: S2 Table — 1) Sigma-Aldrich Chemie (Taufkirchen, Germany); 2) OriGene Technologies (Rockville, USA). (DOCX) [file pone.0180665.s004.docx]

| **Anti-CPAMD8 antibody** | **Recombinant protein epitope signature tag (PrEST) antigen sequence** | **Verified species reactivity** | **Host** | **Clonality** | **Antigen sequence identity to**  **bovine CPAMD8 ortholog** |
| --- | --- | --- | --- | --- | --- |
| HPA031330^1)^ | SDLGLNNITAKALAYGDTNCCRDGRSSKHPEENHADRRVPIGVDHVRRSVMVEAEGVPRAYTYSAFFCPSERVHISTPNKYEFQYVQR | Human | Rabbit | polyclonal | 82% |
| HPA031327^1)^ | YLPSYLSLGSWYSPSQCYLQLQPPSHPLQVGEEAYFSVKSTCPCNFTLYYEVAARGNIVLSG | Human | Rabbit | polyclonal | 95.16% |
| HPA031328^1)^ | FPETWIWHCLNISDPSGEGTLSVKVPDSITSWVGEAVALSTSQGLGIAEPSLLKTFKPFFVDFMLPALIIRGEQVKIPLSVYNYMGTCAEVYMKLSVPK | Human | Rabbit | polyclonal | 81.82% |
| orb182705^2)^ | unknown | Human, cow, dog | Rabbit | polyclonal | unknown |
