# Supplementary material for: Morgagnian cataract resulting from a naturally occurring nonsense mutation elucidates a role of CPAMD8 in mammalian lens development
Source: PLoS One. 2017 Jul 6;12(7):e0180665. doi: 10.1371/journal.pone.0180665 (PMC5500361; doi:10.1371/journal.pone.0180665)
Supplement: S1 Appendix — (DOCX) [file pone.0180665.s005.docx]

***In vitro* production of bovine recombinant CPAMD8 epitope**

Template amplification was done using FastStart *Taq* DNA Polymerase, dNTPack (Roche Diagnostics, Mannheim, Germany) with following mixture: 2U of FastStart *Taq* DNA Polymerase, 200µM of each dNTP, 2mM MgCl2 and 10µM of following primers 150608_Epitop_AK4_AK1_2_F: ATGACGGACCTGGTGAGC and 150608_Epitop_AK4_AK1_2_R: CAATCATGCCCGCTGTGTC. Primers were again designed with NCBI primer-BLAST and synthesized by Sigma-Aldrich (Taufkirchen, Germany). 0.4µl cDNA of 1µg reverse transcription from kidney sample of healthy control was used as template. Cycling conditions were set to 95°C for 10 min, followed by 40 cycles of 95°C for 30 sec, 63°C for 30 sec and 72°C for 60 sec and a final elongation for 5 min at 72°C.

Production of linear expression template was done using RTS 100 *E. coli* LinTempGen Set, His-tag kit (Biotechrabbit, Hennigsdorf, Germany). Overlap regions were added during PCR. The PCR reaction mix at a total volume of 50µl included 3U Phusion® High-Fidelity DNA Polymerase (Finnzymes, ThermoFisher Scientific, Germany), 250µM dNTP, 250nM of the following gene-specific C-terminal His_6_-tag primers: CPAMD8Epifor: CTTTAAGAAGGAGATATACCATGACGGACCTGGTGAGC and CPAMD8EpiHisrev: TGATGATGAGAACCCCCCCCAATCTAGCCCGCTGTGTC, and 7.5mM MgCl_2_. Cycling conditions were 94°C for 4 min, followed by 94°C for 1 min, 63°C for 1 min and 72°C for 1 min for 20 cycles. Final extension was performed at 42°C for 5 min.

PCR amplification control was performed on a 1.5% agarose gel with ethidium bromide.

Regulatory elements and His_6_-tag were added during second PCR step using identical PCR reaction mix as described above and extra T7 Promoter Primer (480nM), T7 Terminator Primer (480nM), C-terminal His_6_-tag DNA (1x) and 4µl of first PCR product as template. Cycling conditions were 94°C for 4 min, 25 cycles of 94°C for 1 min, 63°C for 1 min and 72°C for 1 min, followed by 72°C for 5 min. Quality control of 5µl of the second PCR product was performed on a 1.5% agarose gel with ethidium bromide.

Expression of the protein was done using RTS 100 E. coli HY Kit (Biotechrabbit, Hennigsdorf, Germany) following manufacturer´s protocol.
